# The Role of Natural Low Molecular Weight Dicarbonyls in Atherogenesis and Diabetogenesis

**DOI:** 10.31083/j.rcm2508295

**Published:** 2024-08-20

**Authors:** Vadim Z. Lankin, Alla K. Tikhaze, Mars G. Sharapov, Galina G. Konovalova

**Affiliations:** ^1^Department for Free Radical Biochemistry, E.I. Chazov' National Medical Research Center of Cardiology, Russian Ministry of Health, 121552 Moscow, Russia; ^2^Institute of Cell Biophysics, Russian Academy of Sciences, 142290 Pushchino, Moscow, Russia

**Keywords:** reactive oxygen species (ROS), free radical oxidation (FRO), superoxide anion radical (O_2_^•-^), lipoperoxides (LOOH), oxidative stress, low molecular weight dicarbonyls, malondialdehyde (MDA), glyoxal, methylglyoxal, reactive carbonyl species (RCS), carbonyl stress, low density lipoproteins (LDL), oxidized (LOOH contained) LDL (LOOH-LDL), MDA-modified LDL (MDA-LDL), lectin like oxidized low density lipoprotein receptor 1 (LOX-1), NADPH oxidase 1 (NOX-1), antioxidant enzymes, superoxide dismutase (SOD), glutathione peroxidase (GSH-Px), peroxiredoxines, endotheliocites, apoptosis, atherosclerosis, diabetes

## Abstract

This review summarises the data from long-term experimental studies and literature data on the role of oxidatively modified low-density lipoproteins (LDL) in atherogenesis and diabetogenesis. It was shown that not “oxidized” (lipoperoxide-containing) LDL, but dicarbonyl-modified LDL are atherogenic (actively captured by cultured macrophages with the help of scavenger receptors), and also cause expression of lectin like oxidized low density lipoprotein receptor 1 (*LOX-1*) and nicotinamide adenine dinucleotide phosphate (NADPH) oxidase 1 (*NOX-1*) genes in endotheliocytes, which stimulate apoptosis and endothelial dysfunction. The obtained data allowed us to justify new approaches to pharmacotherapy of atherosclerosis and diabetes mellitus.

## 1. Atherosclerosis as Free Radical Pathology

Denhem Harman was the first scientist to herald the hypothesis that the aging of an organism is caused by the accumulation of molecular lesions resulting froms buildup of the products of free radical reactions [1]. Consequently, he coined the term “free radical disease” in order to signify such age-related pathology as atherosclerosis [2]. However, the first experimental data on the content of free radical oxidation (FRO) products in the regions of atherosclerotic lesions in human aorta were rather contradictory [3, 4]. Not earlier than two decades later the adequate method such as high performance liquid chromatography (HPLC) detected pronounced elevation of the content of lipohydroperoxides (LOOH), which are the primary FRO products, in aortal autopsy samples of atherosclerotic patients [5, 6]. Importantly, HPLC employing the column with chiral phase detected equal amounts of S and R stereoisomers of LOOH in the regions of human aortal atherosclerotic damage attesting to their formation due to spontaneous (non-enzymatic) FRO of unsaturated lipids [6]. Simultaneously, diminished activity of the key antioxidant enzymes such as Cu,Zn-superoxide dismutase (Cu,Zn-SOD) and Se-containing glutathione peroxidase (GSH-Px) was observed in the same areas of atherosclerotic lesions [7, 8]. These data assume that atherosclerosis is characterized by an imbalance between the generation and utilization of FRO products [5, 8, 9]. Based on these results, one could reliably consider atherosclerosis as “free radical pathology” [5].

Significant increases in the levels of primary and secondary products FRO of the lipids were observed in representative epidemiological studies in the blood plasma of probands with diagnosticated atherosclerosis [5, 8]. These data assumed that in atherogenesis, oxidisation of the nanoparticles of the lipid transport system in blood plasma, *i.e.*, the low-density lipoproteins (LDL), which are easily subjected to FRO initiated by metal ions of variable valence and other oxidation inductors [10]. Chemical modification of LDL with acetaldehyde made them “more atherogenic” [11], *i.e.*, capable of binding with the scavenger receptor of macrophages and build up in the vascular wall [11]. In numerous subsequent studies, it was found that oxidised LDL also became “atherogenic” [12, 13, 14, 15, 16, 17, 18].

## 2. Free Radical Peroxidation of Biomembranes and LDL. Malondialdehyde (MDA)-Modified LDL in Atherosclerosis 

FRO of polyene lipids is performed in two stages: initially, the primary products are formed, which are unstable LOOH subjected to subsequent oxidative destruction with formation of the secondary products, *i.e.*, the low molecular weight dicarbonyls [19]. Therefore, the oxidative stress in atherogenesis which is characterised by dramatic LOOH elevation in tissues must inevitably transform into the carbonyl stress accompanied with buildup of such reactive carbonyl species (RCS) as hydroxynonenals and malondialdehyde (MDA) [8, 19]. The aldehyde groups of dicarbonyls can rapidly react with the terminal amino groups of the proteins according to the Maillard reaction resulting in intra- and inter-molecular cross-links capable of modifying the proteins [19]. Although the implication of MDA in chemical modification of apoprotein B-100 in LDL was firmly established [20], the molecular mechanism of oxidative modification of LDL resulting in their ‘atherogenicity’ [10] is still not clear.

In strict terms, the oxidized LDL are those which contain LOOH-acyls in the phospholipids of the outer layer of the particles [10]. Importantly, accumulation of LOOH-acyls in the outer phospholipid monolayer of LDL can change the conformation of apoprotein B-100, the only protein in LDL. Actually, FRO of unsaturated “fluid” acyls of membrane phospholipids results in a dramatic rise of membrane microviscosity [5, 21] due to the displacement of the more polar LOOH acyls into aqueous phase and elevation of the content of saturated ‘solid’ fatty acid residues in the phospholipids resulting in increased membrane rigidity [5, 21]. Evidently, the pronounced changes of such fundamental properties of biomembranes as microviscosity and polarity can possibly modify conformation of peripheral and integral proteins incorporated into the phospholipid bilayer. Specifically, FRO of microsomes is characterized by divergent changes in activity of the membrane-bound enzymes: activity of the oxidation-sensitive enzymes decreases while that of oxidation-resistant enzymes increases [5, 8]. This phenomenon is explained by a physical change in the conformation of these protein molecules caused by a change in the physicochemical properties of membrane lipids. These data suggest that LOOH accumulation in LDL phospholipids may alter the conformation of apoprotein B-100, which may cause changes in the binding efficiency of oxidised LDL with scavenger receptor of macrophages.

After *in vitro* FRO initiation in LDL with diverse inducers such as azo-initiators, H_2_O_2_, superoxide anion radical (O_2_^•-^) generators, metal ions with variable valence, *etc*., elevation of concentration of primary (LOOH) and secondary (MDA) products of lipoperoxidation occurs virtually simultaneously [10].

At the same time, the formation of MDA-modified LDL occurs only after a significant accumulation of MDA in the incubation medium [10]. Thus, the medium in which LDL had been oxidized for a long time contains a mixture of LOOH-containing LDL (LOOH-LDL) and MDA-modified LDL (MDA-LDL) in unpredictable proportion [10]. Therefore, any study of physiological effects of such mixtures of oxidatively modified LDL cannot establish what kind of products of lipid FRO provoke ‘atherogenic’ modification of LDL particles. By employing the homogenous preparation C-15 of lipoxygenases of rabbit reticulocytes known to catalyze oxidation of polyene acyls of phospholipids in the lipid-protein supramolecular complexes (LDL included, [22]), we could obtain the LOOH-LDL without admixture of MDA-LDL [23]. Simultaneously, incubation of LDL with MDA yielded MDA-modified LDL without admixture of LOOH-LDL [23]. Examination of atherogenicity of modified LDL (*i.e.*, efficacy of LDL captured by cultured human macrophages) unequivocally proved that not oxidized (LOOH-LDL), but exclusively MDA-LDL bind with the scavenger receptors of macrophages [23]. Thus, expressly LDL particles that are modified with natural dicarbonyls but not the oxidized LDL (*i.e.*, LOOH-LDL) are effectively captured in vascular wall cells to be accumulated in the lipid vacuoles [23]. As a consequence of buildup of exogenous lipids, the macrophages and pleomorphic smooth muscle cells convert into so-called foam cells, which form the lipidosis regions, where the primary pre-atherosclerotic lesions to vascular wall develop [5, 8]. These facts do not merely refine the current terminology, but they principally renovate it due to substantiation of certain molecular mechanisms of atherogenic modification of LDL particles with implication of natural low molecular weight carbonyl compounds. The representative epidemiologic studies corroborated the view that atherogenic (cholesterol-rich) LDL particles also are and the MDA-modified ones [24]. Consequently, the carbonyl modification of LDL particles promotes effective delivery of cholesterol into the vascular wall [24]. Moreover, the novel data show that enhanced accumulation of MDA-modified LDL is typical of the patients with certain mutations of apoprotein B-100, which means that carbonyl modification of LDL can be genetically determined [25]. Endothelial dysfunction induced by oxidative stress also plays an important role in the development of cerebrovascular diseases, particularly in stroke [26]. An important role in the progression of these diseases is associated with the activation of nicotinamide adenine dinucleotide phosphate (NADPH)-oxidase generating O_2_^•-^, however, the role of LOX-1 and dicarbonyl-modified LDL in this process has not been studied to date [26].

## 3. Dicarbonil-Dependent Inhibition of Antioxidant Enzymes in Atherosclerosis

Similar to apoprotein B-100, the protein molecules Cu,Zn-SOD and GSH-Px are modified during the accumulation of MDA resulting in the inhibition of their activity [27, 28] because of conformational alterations in the structure of the active center [29]. Evidently, the dicarbonyl-dependent inhibition of activity of the antioxidant enzymes during atherogenesis should stimulate oxidative stress. Hence, the development of oxidative stress (hallmarked by LOOH buildup) and the following carbonyl stress (reported by MDA accumulation) in atherogenesis lead to the formation and accumulation of dicarbonyl-modified LDL, which are the key factors provoking the pre-atherogenic lesions to vascular wall [30].

## 4. The Role of Dicarbonil-Modified LDL in Diabetogenesis

It is common knowledge that diabetes mellitus is a risk factor for atherosclerosis, and diabetes promotes its rapid progress; however, the majority of diabetic patients die of vascular incidents [31, 32, 33]. Nevertheless, the available literature does not comprehensively explain the pathophysiological mechanisms associated with these phenomena. A rather long time ago, a hypothesis was advanced on the important role of FRO in the pathogenesis of diabetes mellitus [34]. Importantly, diabetes mellitus is characterized by the development of not oxidative but carbonyl stress [35] characterised by the accumulation of not MDA, but RCS, formed during oxidative transformation of glucose, such as glyoxal (MDA homolog) and methylglyoxal (MDA isomer) [35, 36, 37]. Glyoxal is formed in the process of glyoxylation during autoxidation of glucose and other six-carbon carbohydrates, whereas methylglyoxal is mainly produced by glycolysis during the enzymatic oxidation of glucose with the generation of triozophosphates [35, 36, 37]. In addition, glyoxal and methylglyoxal can be produced non-enzymatically when hydroperoxyl free radicals attack glucose [38, 39] or its derivatives [40]. During co-oxidation of LDL in the presence of glucose in concentrations characteristic of its level in the blood of patients with insulin-independent (type II) diabetes, a dramatic increase in the rate of FRO of LDL occurs in parallel with the augmented generation of O_2_^•-^ [41]. During the Maillard reaction, *i.e.*, the interaction of methylglyoxal with terminal amino groups of apoprotein B-100, generation of O_2_^•-^ is also possible [42]. Therefore, in contrast to atherogenesis, diabetogenesis is characterized by the initial development of the carbonyl stress manifested by RCS accumulation, while oxidative stress is the secondary process, provoked at the later stages of the disease by reactive oxygen species (ROS) generated in the above reactions. Accordingly, in diabetogenesis, one should differentiate the stages of carbonyl stress development and the subsequent oxidative stress manifested by the accumulation of diverse oxidation products. Accumulation of glyoxal and methylglyoxal in the blood plasma of patients with diabetes mellitus was documented experimentally [36, 37]. In patients with insulin-independent (type II) diabetes, the carbonyl modification of LDL is essentially increased [41] in parallel with a pronounced decrease of activity of erythrocytic Cu,Zn-SOD and GSH-Px [28, 43], which are the typical hallmarks of carbonyl stress. At the same time, the oxidative stress in diabetic patients is manifested by a decrease in the length of telomeres in the nucleated blood cells [43] as well as by the rise of the blood and urine levels of 8-hydroxy-2′-deoxyguanosine (the final product of oxidative DNA destruction) in patients with insulin-independent (type II) diabetes [43]. It is worth noting that 8-hydroxy-2′-deoxyguanosine is a widely accepted biomarker of oxidative stress [44]. The enhanced level of LOOH-LDL [41] in the blood of patients with insulin-independent (type II) diabetes also attests to the possibility that the secondary induction of oxidative stress can occur in diabetogenesis. Pronounced elevation of blood concentrations of glyoxal and methylglyoxal in patients with type II diabetes [36, 37, 45] can provoke modification of LDL, which will be recognised by the scavenger receptors in macrophages with subsequent development of lipoidosic lesions in the vascular wall. Specifically, modification of LDL with glyoxal essentially augments the receptive capture of LDL by macrophages [35]. Based on the above data, we advanced a hypothesis on the similar molecular mechanism underlying lesions to vascular wall in atherosclerosis and diabetes mellitus, which assumes enhancement of chemical modification of apoprotein B-100 in LDL with dicarbonyls that are accumulating during FRO of the lipids in atherosclerosis or oxidative transformations of glucose molecules in diabetes mellitus [41, 46]. This hypothesis reliably explains the reasons for atherogenesis stimulation in diabetes and the fact that diabetes elevates the risk of atherosclerosis [41, 46].

## 5. Lectin-Like Oxidized LDL Receptor LOX-1 and Endothelial Dysfunction

Recent studies have shown that oxidized LDL plays an important role in the onset of endothelial dysfunction [30, 46, 47, 48, 49, 50, 51]. It is hypothesized that endothelial lectin-type oxidized LDL receptor 1 (LOX-1) binds with oxidized LDL thereby triggering expression of NADPH oxidase (NOX-1), which generates O_2_^•-^ which is responsible for the damage to endotheliocytes [30, 46, 52]. It was established that powerful expression of *LOX-1* and *NOX-1* in human endotheliocytes is caused by culturing the cells with dicarbonyl-modified (MDA-, glyoxal-, and methylglyoxal-modified) LDL [53], the greatest expression of *LOX-1* and *NOX-1* being provoked by MDA-modified LDL. Simultaneously, a compensatory reaction was revealed in the endothelial cells based on the expression of the genes responsible for the biosynthesis of the antioxidant enzymes such as SOD, GSH-Px, and peroxiredoxins [53]. Despite these processes, the expression of genes relating to the key factors of apoptosis (BCL2-associated X protein (BAX), caspase 9, and caspase 3) elevates, which finally stimulates damage and apoptosis in endotheliocytes [53]. Thus, the initial stages in the development of endothelial dysfunction known to play the leading role in atherogenesis and diabetogenesis directly depend on the formation of non-oxidized (LOOH-containing) but dicarbonyl-modified LDL [53]. In the end, O_2_^•-^-dependent lesions of endotheliocytes provokes stimulation of apoptosis and the death of these cells [30, 46, 52, 53], which in turn, alleviates invasion of dicarbonyl-modified LDL into the vascular wall.

## 6. Antioxidant Enzymes of Endotheliocytes. Their Inhibition by Natural Low Molecular Weight Dicarbonyls

The enzymatic antioxidant system in endotheliocytes is formed predominantly by a special class of enzymes, *i.e.*, peroxiredoxins [54], which similar to Cu,Zn-SOD and GSH-Px [28] are rather sensitive to the inhibitory action of low molecular weight dicarbonyls accumulating during oxidative and carbonyl stresses [55]. It is beyond any doubt that suppression of activity of peroxiredoxins weakens the antiradical defense of endothelial cells thereby promoting their damage and endothelial dysfunction. The available data suggest that the generation of dicarbonyl-modified LDL is the key factor in the development of endothelial dysfunction, which is the leading process in atherogenesis and diabetogenesis.

## 7. Free Radicals as Promoters of Endothelial Glycocalyx Fragmentation

**Fig. 1.**
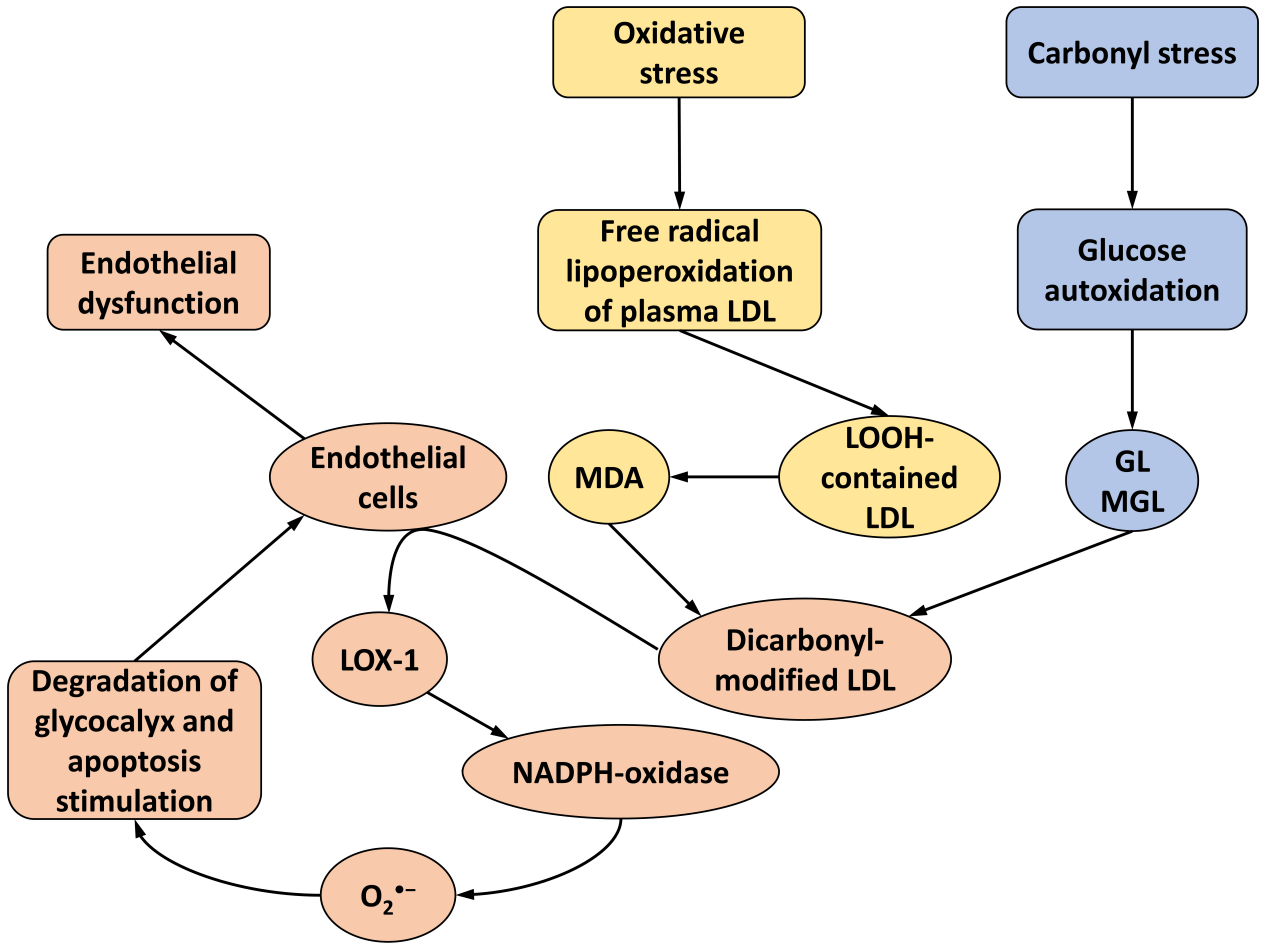
**Main stages of vascular wall damage under oxidative/carbonyl stress and development of endothelial dysfunction in the process of atherogenesis/diabetogenesis under the actions of dicarbonyl-modified LDLs according to the literature and our results**. LDL, low-density lipoproteins; MDA, malondialdehyde; GL, glyoxal; MGL, methylglyoxal; LOOH-contained LDL, lipohydroperoxydes-contained LDL (see explanation in the text); LOX-1, lectin like oxidized low density lipoprotein receptor 1; NADPH, nicotinamide adenine dinucleotide phosphate.

Clearly, a lesion to endothelial glycocalyx should precede the development of endothelial dysfunction. Glycocalyx is the protective layer composed of macromolecules such as proteoglycans and glycoproteins, which cover the luminal face of endotheliocytes [56, 57]. A lesion to glycocalyx is considered as the earliest stage in the damage to vascular wall in diverse pathologies [58, 59, 60, 61]. Now it is a common knowledge that glycocalyx controls vascular permeability [62] and adhesion of the blood formed elements on the outer face of endotheliocytes [63, 64]. Moreover, glycocalyx protects the endothelium against a moiety of damaging factors such as viruses, proinflammatory cytokines, and ROS [65, 66]. Presumably, namely, glycocalyx is the barrier that prevents penetration of atherogenic LDL into the subendothelial space of the vascular wall [67]. Importantly, thinning of glycocalyx due to its fragmentation was observed in the process of O_2_^•-^ hyperproduction (“oxidative burst”) during ischemia and/or ischemia-reperfusion injury [68, 69, 70]. It is worthy to note that a lesion to glycocalyx was revealed when the blood plasma level of oxidized LDL increased [71, 72], which can be explained by the enhanced production of O_2_^•-^ due to the expression of LOX-1 and NADPH oxidase [30, 46, 53]. These facts are evidence that oxidatively modified LDL (most probably, the dicarbonyl-modified ones) generated during oxidative and carbonyl stresses are the key factors in the outbreak and the progress of endothelial dysfunction. The integrity of glycocalyx should prevent the development of athero- and diabetogenesis, whereas damage to glycocalyx is probably the first stage in the atherosclerotic lesion to the vascular wall. The previously described (see review sections 2–7) processes leading to the development of endothelial dysfunction are presented in the following scheme (Fig. 1).

## 8. Reasons for Failure in Using LDL Protection against FRO by Natural Antioxidants

Based on the above premise, it seems logical to employ antioxidants to inhibit lipoperoxidation of LDL. With this aim in mind, some clinical studies tested safe natural antioxidants such as vitamin E (α-tocopherol, α-TOH). In contrast to rather promising results obtained in the animals with experimental atherosclerosis, the clinical trials of the action of antioxidants (α-TOH predominantly) in cardiovascular diseases were not so unequivocal [30, 73, 74, 75, 76]. However, it should be noted that in most clinical studies, the use of vitamin E as an antioxidant was not reasonable. One should take into consideration that vitamin E is the dosage form, in which α-TOH is included as the ethers of organic acids (acetate or succinate), so it is not an antioxidant because of the blocked OH-group. In the intestine, the esterified α-TOH can be hydrolyzed, although there are no data on the effectiveness of this process in patients with ischemic heart disease or atherosclerosis. Thus, there is no firm evidence that α-TOH was available in the patients’s organisms in the phenolic form capable to exert its antioxidant effects. Moreover, similar to other phenolic antioxidants, α-TOH does not block FRO of the lipids completely, but it only decreases FRO intensity due reduction of the hyperactive lipid hydroxyperoxyl (LO_2_^•^) and alkoxy (LO^•^) radicals with production of low-activity radical inhibitor (tocoferoxyl radical – α-TO^•^) according to the following reaction:

$${\text{LO}}_{2}^{∙}\,\left({\text{LO}}^{∙}\right)+\alpha \,\text{-TOH}\to \text{LOOH}\,\left(\text{LOH}\right)+\alpha \,{\text{-TO}}^{∙}.$$

Under pronounced accumulation of α-TO^•^ radicals, which can occur when antioxidants are administered in high doses, these radicals can induce FRO. Therefore, the antioxidant effect can transform into the pro-oxidant one [77]. In other words, the concentration-dependent inversion of the antioxidant effect is possible, which was observed both *in vitro * [78] and *in vivo * [77]. Similar to other liposoluble vitamins, α-TOH is transported within the hydrophobic lipid core of LDL particles [79], although defense of circulating LDL against FRO is performed not by α-TOH, but by the reduced (phenolic) form of coenzyme Q_10_ [80, 81, 82, 83]. Remembering that one LDL particle contains no more than 1-2 molecules of coenzyme Q_10_ per about 800 molecules of FRO substrate, *i.e.*, unsaturated phospholipids [84], effective inhibition of FRO in LDL by this antioxidant is possible only with its efficient reduction (biogeneration), which occurs, probably, with the participation of radical intermediates α-TOH and ascorbate [85, 86, 87, 88]. Administration of α-TOH in high doses produces no effect on LDL oxidability in patients [83], which means that the use of α-TOH to inhibit the oxidability of LDL in clinical trials is not sufficiently substantiated both theoretically and experimentally. At the same time, coenzyme Q_10_ effectively suppresses FRO of liver biomembranes *in vitro * [89] and LDL particles *in vivo * [83]. The data of clinical trials with high doses of α-TOH revealed no apparent positive clinical effects, although at the same time, there were no detrimental consequences of this antioxidant therapy [30, 46]. When analyzing these studies, some authors allege without sound reasons that the antioxidants exert negative clinical action [74, 75], although it is incorrect to consider the absence of an effect as the negative action. From our viewpoint, it is unacceptable to generalise seemingly “negative” data harvested with some antioxidants such as α-TOH [8, 30, 46] onto a rather diverse group of antioxidants that consists of compounds with different structures and mechanisms of action. Enhanced effectiveness in the protection of LDL against oxidation is typical not only for coenzyme Q_10_ [83] but other phenolic antioxidants such as non-toxic synthetic antioxidant probucol [90, 91, 92] which has been shown to effectively inhibit free radical peroxidation of LDL *in vivo * [41, 83, 92].

## 9. Perspectives of Pharmacotherapy Aimed at Enhancing the Utilisation of Low Molecular Weight Natural Dicarbonyls

The data summarized in this review suggest that to suppress atherogenesis and prevent endothelial dysfunction, it is necessary to inhibit not only accumulation of the primary products (LOOH) in LDL, but also to suppress the buildup of secondary FRO products, *i.e.*, low molecular weight dicarbonyls. Theoretical substantiation and experimental confirmation of the leading role of secondary products of free radical peroxidation of lipids and products of oxidative transformation of six-atom carbohydrates in the development of endothelial dysfunction in atherosclerosis and diabetes mellitus dictates fundamentally new approaches to pharmacotherapy of these diseases. The main emphasis should be placed on the search for nontoxic compounds that can act as scavengers of natural dicarbonyls, such as MDA, glyoxal and methylglyoxal [93, 94]. Such investigations are already underway, with simple compounds such as glucosamine, taurine, histamine, pyridoxamine, etc. shown to be effective in model systems [95, 96, 97, 98]. Importantly, there are already examples of the successful use of natural dicarbonyl scavengers in clinical trials [99, 100]. Now positive examples are available, which demonstrate the effective inhibition of FRO intensity by the scavengers of dicarbonyls such as biguanides [41, 101] and imidazole-containing peptides [93, 102, 103]. There are also positive examples which demonstrate the effective inhibition of FRO intensity with dicarbonyl scavengers such as biguanides [41, 99, 100] and imidazole-containing peptides in clinical investigations [102, 103]. Specifically, the use of biguanides pronouncedly suppresses the symptoms of oxidative and carbonyl stresses in patients with diabetes mellitus, even without intake of any antioxidants (so-called “quasi-antioxidant effect”) [41]. There are data that hypolipidemic therapy with inhibitor of proprotein convertase subtilisin/kexin type 9 (PCSK9), which activates utilization of cholesterol-rich LDL in the liver, simultaneously lowering the level of MDA-modified LDL in blood plasma [104]. In such therapy, the kinetics of the reduction of LDL and MDA-modified LDL levels virtually coincide attesting to the predominant utilization of oxidatively modified LDLs, indicating why the use of PCSK9 in pharmacotherapy of atherosclerosis seems to be so promising [104]. Determination of the levels of soluble LOX-1, the fragments of glycocalyx, 8-hydroxy-2′-deoxyguanosine and other indices of oxidative and/or carbonyl stress is rather reasonable because they can be viewed as supplementary biomarkers to diagnose and control therapeutic effectiveness in atherosclerosis and diabetes mellitus. Preventive cardiology should aim to prevent the negative consequences of LDL oxidative modification, since dicarbonyl-modified LDL plays a key role in the molecular mechanisms of atherogenesis and diabetogenesis described in this review.

## 10. Conclusions

The review supports the authors’ experimentally proven idea that not “oxidized”, *i.e.*, LOOH-containing LDL particles in phospholipids of the outer layer, but LDL particles chemically modified by low molecular weight natural dicarbonyls are atherogenic and capable of inducing endothelial dysfunction. The authors of numerous experimental studies obtaining “oxidized” LDL by multi-hour initiated FRO inevitably use a mixture of truly oxidised (LOOH-containing) LDL and MDA-modified LDL formed in the incubation medium. The atherogenic effect of these conditions, as well as stimulation of endothelial dysfunction, is caused exclusively by dicarbonyl-modified LDLs. The hypothesis put forward here allows a satisfactory explanation for the cause of the progression of atherosclerotic lesions in the vascular wall in the presence of diabetes mellitus, as well as proposing new approaches for the pharmacotherapy of atherosclerosis and diabetes.
